# Supplementary material for: Betel Nut Chewing Is Associated with the Risk of Kidney Stone Disease
Source: J Pers Med. 2022 Jan 18;12(2):126. doi: 10.3390/jpm12020126 (PMC8879579; doi:10.3390/jpm12020126)
Supplement: Supplementary file 1 [file jpm-12-00126-s001.zip › jpm-1423116-supplementary.pdf]

Original Article

Betel nut chewing is associated with the risk of kidney stone disease

Chun-Kai Chang[1,2,3], Jia-In Lee[4], Chu-Fen Chang[5], Yung-Chin Lee[1,2,3], Jhen-Hao Jhan[1,2,3], Hsun-Shuan Wang[1,2,3], Jung-Tsung Shen[1], Yao-Hsuan Tsao[1,2,3], Shu-Pin Huang[2,3,6,7,8], Jiun-Hung Geng [1,2,3,7,9,*]

1 Department of Urology, Kaohsiung Municipal Siaogang Hospital, Kaohsiung, Taiwan

2 Department of Urology, Kaohsiung Medical University Hospital, Kaohsiung, Taiwan

3 Kaohsiung Medical University, Kaohsiung, Taiwan

4 Department of Psychiatry, Kaohsiung Medical University Hospital, Kaohsiung Medical University, Kaohsiung, Taiwan

5 Department of Physical Therapy, Tzu Chi University, Hualien, Taiwan, R.O.C.

6 Department of Urology, Faculty of Medicine, College of Medicine, Kaohsiung Medical University, Kaohsiung, Taiwan

7 Graduate Institute of Clinical Medicine, College of Medicine, Kaohsiung Medical University, Kaohsiung, Taiwan

8 Ph.D. Program in Environmental and Occupational Medicine, College of Medicine, Kaohsiung Medical Uni-versity, Kaohsiung, Taiwan

9 Research Center for Environmental Medicine, Kaohsiung Medical University, Kaohsiung, Taiwan

*Correspondence: u9001090@hotmail.com

Supplementary Table S1. Relative risk for kidney stone disease in a subgroup analysis for subjects without a history of hypertension, DM, dyslipidemia, gout, and obesity (BMI≧30kg/m2) (N = 28,481)

	No. of KSD / No. of subjects (%)	Odds ratio (95% CI)	*P* value
Never chewer	1,901/24,377 (7.8)	1 (ref)	
Current or ever chewer	361/4,104 (8.8)	1.140 (1.014 to 1.283)	0.029

Abbreviations: KSD : Kidney stone disease; CI : Confidence interval.
